# Are we able to reduce the mortality and morbidity of oral cancer; Some considerations

**DOI:** 10.4317/medoral.18486

**Published:** 2012-12-10

**Authors:** Isaäc van der Waal

**Affiliations:** 1 VU University Medical Center (VUmc)/Academic Centre for Dentistry Amsterdam (ACTA) Department of Oral and Maxillofacial Surgery and Oral Pathology; 2 ……..; 3 ….

## Abstract

Oral cancer makes up 1%-2% of all cancers that may arise in the body. The majority of oral cancers consists of squamous cell carcinomas. Oral cancer carries a considerable mortality rate, being mainly dependent on the stage of the disease at admission. Worldwide some 50% of the patients with oral cancer present with advanced disease. There are several ways of trying to diagnose oral cancer in a lower tumor stage, being 1) mass screening or screening in selected patients, 2) reduction of patients’ delay, and 3) reduction of doctors’ delay. Oral cancer population-based screening (“mass screening”) programs do not meet the guidelines for a successful outcome. There may be some benefit when focusing on high-risk groups, such as heavy smokers and heavy drinkers. Reported reasons for patients’ delay range from fear of a diagnosis of cancer, limited accessibility of primary health care, to unawareness of the possibility of malignant oral diseases. Apparently, information campaigns in news programs and TV have little effect on patients’ delay. Mouth self-examination may have some value in reducing patients’ delay. Doctors’ delay includes dentists’ delay and diagnostic delay caused by other medical and dental health care professionals. Doctors’ delay may vary from almost zero days up to more than six months. Usually, morbidity of cancer treatment is measured by quality of life (QoL) questionnaires. In the past decades this topic has drawn a lot of attention worldwide. It is a challenge to decrease the morbidity that is associated with the various treatment modalities that are used in oral cancer without substantially compromising the survival rate. Smoking cessation contributes to reducing the risk of oral cancers, with a 50% reduction in risk within five years. Indeed, risk factor reduction seems to be the most effective tool in an attempt to decrease the morbidity and mortality of oral cancer.

** Key words:**Oral cancer, early diagnosis, quality of life.

## Introduction

Oral cancer makes up approximately 1%-2% of all cancers that may arise in the body. The great majority of oral cancers consists of squamous cell carcinomas. The remaining cancer types include malignant salivary gland tumors, sarcomas of the soft tissues and the jaw bones, melanoma, malignant odontogenic tumors, lymphoreticular malignancies, and metastases from tumors located elsewhere in the body.

Oral cancer carries a considerable mortality rate, being mainly dependent on the stage of the disease at admission. The five-year survival rate of stage I cancer, including the various subsites such as borders of the tongue, floor of the mouth, cheek, and gums amounts approximately 80%, while the five-year survival of patients with advanced disease (stages III/IV) is approximately 20%. Worldwide some 50% of the patients with oral cancer present with advanced disease (1). Early diagnosis, therefore, seems key. Even more important is to prevent oral cancer, which can be achieved mainly by cessation of heavy tobacco and alcohol consumption.

In a previous paper on early diagnosis of oral cancer it has been mentioned that the adjective “early” can be used in three ways, being 1) early in the process of carcinogenesis, 2) early in the meaning of a relatively small size of the cancer at the time of diagnosis, and 3) early in the meaning of a short interval between the time of symptoms and the time of diagnosis (2).

At present, there are no serological markers or any other laboratory studies that are helpful in detecting primary oral squamous cell carcinoma (OSCC) in a stage where there is no measurable tumor or precursor yet (3). Besides, displacing a diagnosis of cancer to an earlier stage of the carcinogenesis may prolong the survival time without actually influencing the time of death. This pitfall has been termed “lead-time-bias” (4).

As mentioned earlier, small stage OSCC carries a better survival rate than large stage OSCC. It is generally believed that patients with a short diagnostic delay (patients’ and doctors’ delay together) carry a better prognosis than those with a long diagnostic delay. However, the assumed better survival rate has not been confirmed in a few studies on this subject from the United Kingdom (5,6). Furthermore, early detection and treatment of oral precancerous lesions, particularly leukoplakia and erythroplakia, may not truly prevent the future development of OSCC at the siteof the treated lesion or elsewhere in the oral cavity (7).

## Reducing the mortality of oral cancer by detection oral cancer in a less advanced stage

There are several ways of trying to diagnose oral cancer in a lower tumor stage, being 1) mass screening or screening in selected patients, 2) reduction of patients’ delay, and 3) reduction of doctors’ delay.

-Oral cancer population-based screening (“mass screening”) programs do not meet the epidemiological guidelines for a successful outcome and are not considered to be cost-effective in its current forms (8). There may be some benefit when focusing screening programs on high-risk groups, such as heavy smokers and heavy drinkers (9), patients with previous cancer in the head and neck area (10), and patients with previous cancer outside the head and neck area (11).

In a study in Japan the results of oral screening as an integral part of general health screening in adults over 40 years of age have been reported (12). Some 26% of the population participated in the general health screening program and almost all of these entered the oral cancer screening program performed by dentists. The results of the oral screening are shown in ( Table 1). These results do not allow to draw any conclusions about the possible positive effect of the oral cancer screening program on morbidity and mortality. It merely shows that subjects who participate in a general health screening program almost without exception want to participate in an oral cancer screening program. Furthermore, no information has been provided in the Japanese study about the costs involved in the general health and oral cancer screening program.

**Table 1:**
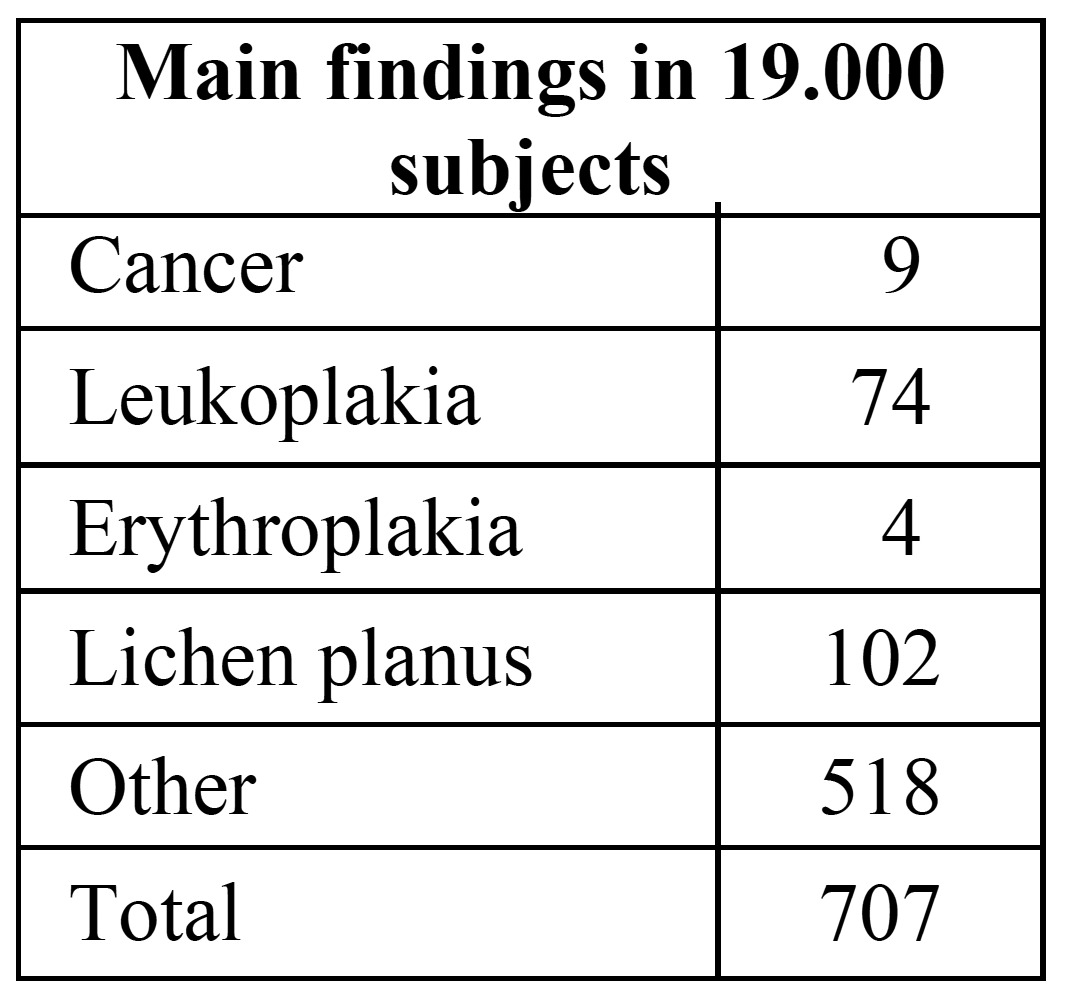
Oral cancer screening as part of a free annual general health screening program in Japan (slightly modified) (12).

-Reduction of patients’ delay may reduce morbidity and may decrease the mortality rate. The average patients’ delay in oral cancer diagnosis is approximately three months (13). Reported reasons for such delay range from fear of a diagnosis of cancer, limited accessibility of primary health care for patients with a low social economic status to, above all, unawareness of the possibility of malignant disease in case of a symptomatic oral lesion (14).

Apparently, information campaigns in news programs and TV have little effect on patients’ delay (15). Few educational materials for the general public are available that are specifically directed to oral cancer and those that are available may be written at too a high grade level for the general public (16). In 2001 a leaflet on oral cancer directed at dental patients has been published ( Table 2) (17). To the best of our knowledge the effectiveness of this leaflet has not been examined yet. Rogers et al. reported the results of a survey among 71 oral and oropharyngeal cancer patients (18). A nonhealing ulcer or sore was reported as the most common first symptom; some 50% interpreted their symptoms as something minor, not being aware of oral cancer. Altogether, there is a lack of evidence that any public intervention has a measurable impact on oral cancer incidence and morbidity other than tobacco and alcohol consumption control (19).

**Table 2:**
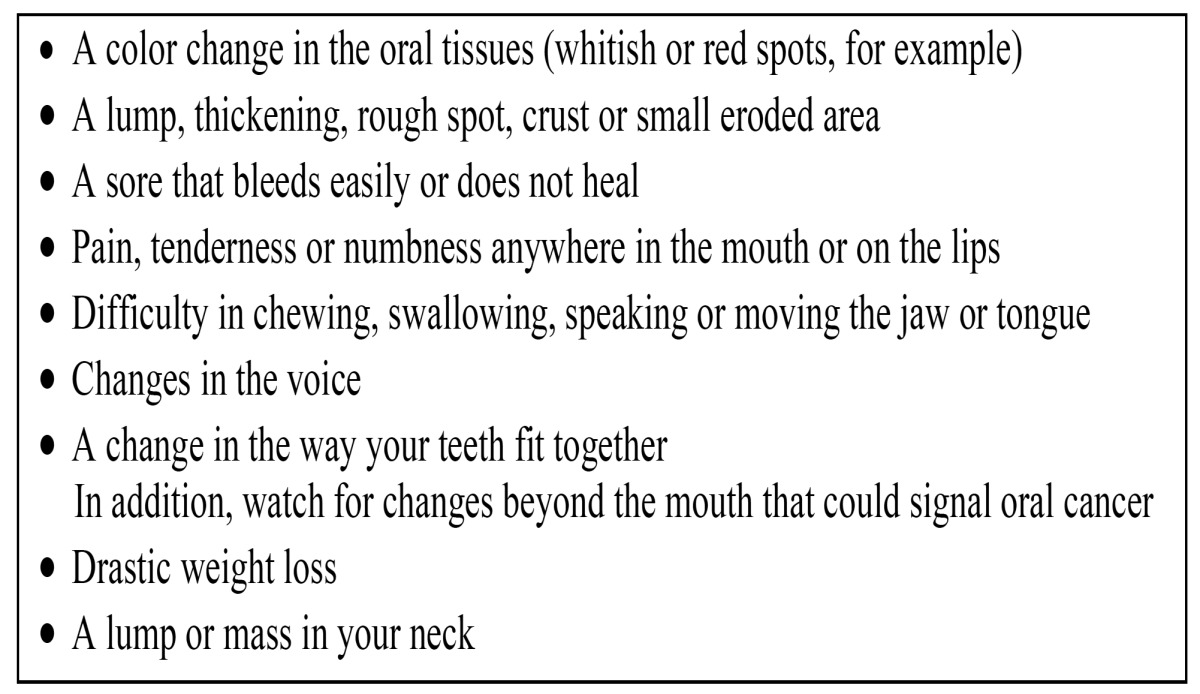
Oral cancer: what to look for? A leaflet for dental patients (17).

-Mouth selfexamination

In an Editorial by Sarode et al. the potential role of mouth self-examination has been emphasized as a method for reducing the morbidity and mortality rate of oral cancer (20). In a study in India, using a brochure describing the risk factors of oral cancer and precancer, and also showing the appearance of these lesions by photographs, 247 patients out of 8.000 patients who practiced mouth self-examination reported to the clinics (21). The findings in these 247 patients have been presented in ( Table 3). The authors advised to further examine the possible value of mouth self-examination in a randomized controlled study, including much larger study groups and observing a follow-up period of at least ten years before being able to demonstrate a possibly significant reduction of the mortality rate. In a differently structured study, also conducted in India, the reliability of the results of mouth self-examination in some 34.000 persons were subsequently checked by health care workers (22). The results are shown in ( Table 4). Apparently, mouth self-examination had a low sensitivity of 18%, while the specificity was almost 100%. A somewhat similar experience was observed in another study in the United Kingdom (23).

-Reduction of doctors’ delay in the diagnosis of oral cancer

**Table 3:**
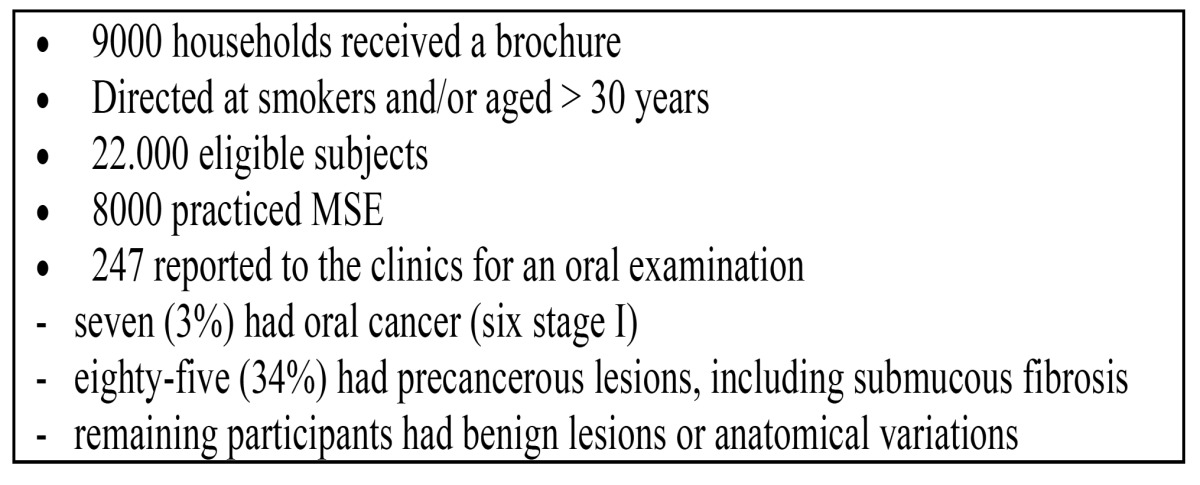
Mouth self-examination in India (21).

**Table 4:**
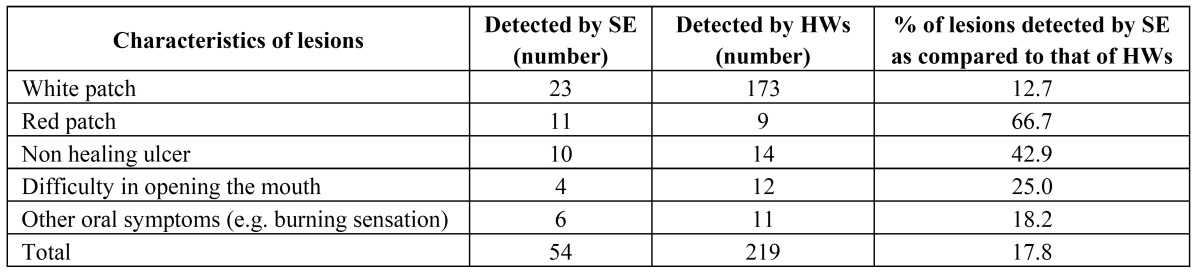
Characteristics of oral lesions by self-examination and health worker examination (n=34.000) (22).

Doctors’ delay includes dentists’ delay and also diagnostic delay caused by other medical and dental health care professionals. A dentist may not encounter an average of more than 5-10 patients with oral cancer during his professional life, while this number may be even less for medical practitioners. Also in view of the rather nonspecific symptoms and the diversity of clinical presentations of oral cancer, it should be no surprise that there is often a considerable delay in suspecting malignancy in case of oral cancer. Such delay may vary from almost zero days up to more than six months with a mean of three to five weeks (13,24). Doctors’ delay of more than five weeks occurs significantly more often in patients under the age of 40 years. Klosa et al. reported a discrepancy in the dentists opinions and practices in routine oral examination for cancerous and precancerous lesions (25).

Not surprisingly, it has been shown by Patton et al. that dental health professionals are more adequately trained to perform oral cancer examination than physicians (26). Physicians, indeed, felt a need for improvement of their oral examination skills. In a study from the United Kingdom medical professionals were questioned about possible barriers to conduct oral examinations for cancer (27). Barriers mentioned to perform such examinations were lack of training, lack of knowledge, lack of equipment, lack of time, and the notion that dentists are the ones who are primarily responsible for oral cancer detection. The latter notion was also mentioned in a study among primary health care professionals (28).

## Reducing the morbidity of oral cancer treatment

Morbidity of oral cancer treatment may be related to surgery, radiotherapy, chemotherapy and other treatment modalities and treatment related modalities, including dental rehabilitation. Usually, morbidity is measured by quality of life (QoL) questionnaires. Furthermore, QoL includes not only physical and mental health, but also factors such as family and leisure activities. The WHO has defined QoL as “Individual perceptions of their position in the context of the culture and value systems in which they live and in relation to their goals and concerns” (29).

In a study among 561 patients treated by surgery for oral and oropharyngeal cancer six groups were identified arranging from T1T2 oral cancer, no free flap, no RT to T3T4 oral cancer, free flap + RT and, as a separate group, oropharyngeal cancer (30). The items that have been scored are listed in  Table 5. The questionnaires have been completed at the time of admission and at 6 and 12 months after completion of treatment. Not surprisingly, the patients who do best are those with small oral cancers, not needing free-flap surgery and not having radiotherapy. This finding was confirmed in a study in Spain (31); in this latter study patients less than 65 years obtained higher QoL scores. De Wit et al. studied donor site morbidity of the fasciocutaneous radial forearm flap (32). Donor site morbidity measured by functional tests was limited but subjective self ratings revealed complaints regarding cosmetics, sensibility and forearm disability.

**Table 5:**
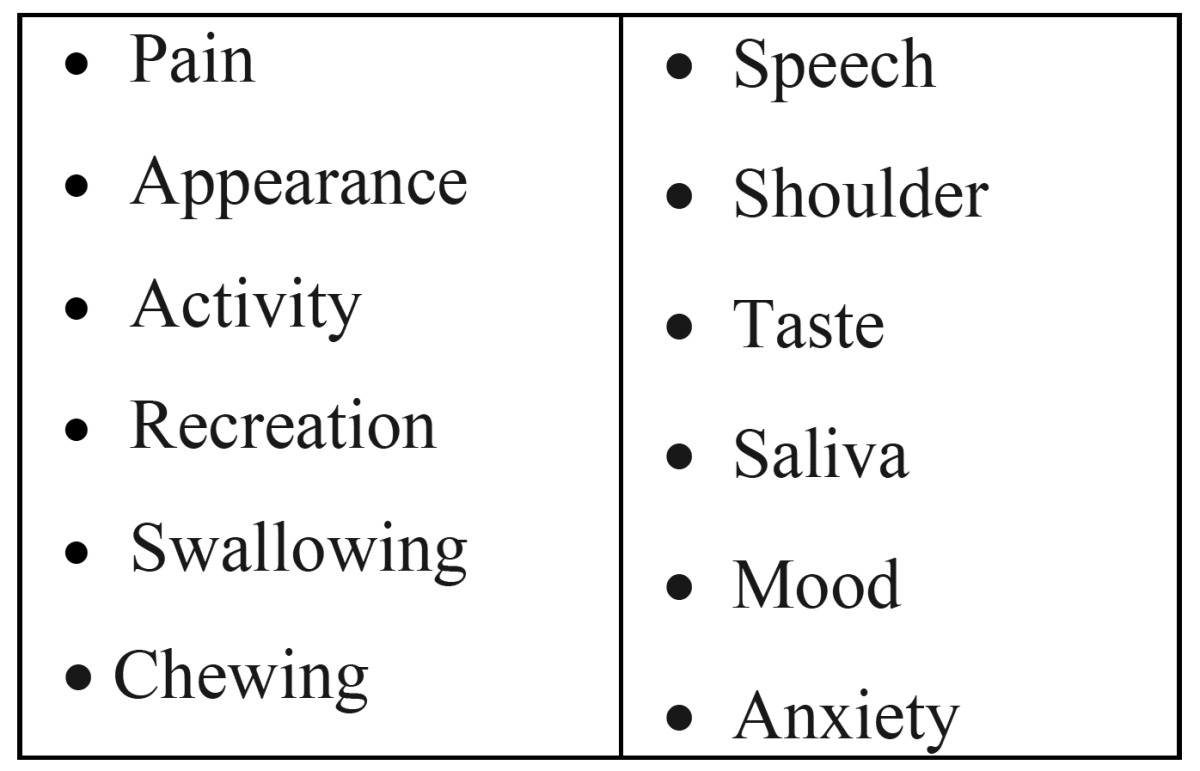
Items of a QoL questionnaire used in patients treated by primary surgery for oral and oropharyngeal cancer (30).

In a study in the United Kingdom it was shown that cancer of the head and neck (HNC) has a serious impact on financial aspects of patients’ life. In a survey in The Netherlands it was shown that HNC survivors return to work within six months after treatment (33); oral dysfunction, loss of appetite, deteriorated social functioning, and high levels of anxiety were barriers for HNC survivors to return to work after treatment. In a study by Borggreven et al. is was shown that comorbidity has a major impact on patients treated for HNC (34). In Verdonck’s et al study is was shown that distress is often present in spouses and patients after treatment for HNC (35). Distress in patients was related to the presence of a feeding tube, speech and swallowing problems, less social contacts, a passive style of coping, and nonexpression of emotions. Handschel et al. emphasized the impact of psychological treatment in HNC patients (36).

Langendijk et al. reported on the late treatment-related toxicity on quality of life among patients with HNC treated with radiotherapy. It was concluded that the development of new radiation-induced delivery techniques should not only focus on reduction of the dose to the salivary glands, but also to anatomic structures that are involved in swallowing (37).

It is a challenge to decrease the morbidity that is associated with the various treatment modalities that are used in oral cancer without substantially compromising the survival rate. On the other hand, improving the survival rate by extended treatment may significantly increase the morbidity and, thereby, influence the quality of life in a negative way.

## Prevention

It is obvious that cessation of tobacco and alcohol use results in a lower incidence of oral cancer. Smoking cessation contributes to reducing the risk of oral cancers, with a 50% reduction in risk within five years (38); ten years after smoking cessation the risk approaches that for life-long nonsmokers.

There are numerous reports on cessation of smoking programs. An interesting overview of these studies is provided by Maillet et al (39). Tobacco cessation counseling can be performed by dentists and also by dental auxiliary personnel, such as dental hygienists (39). In Maillet’s et al study, the results have not been very encouraging, as shown in  Table 6. Only 2% of the patients stopped permanently using tobacco. The authors announced to improve their program and to again evaluate the results.

**Table 6:**
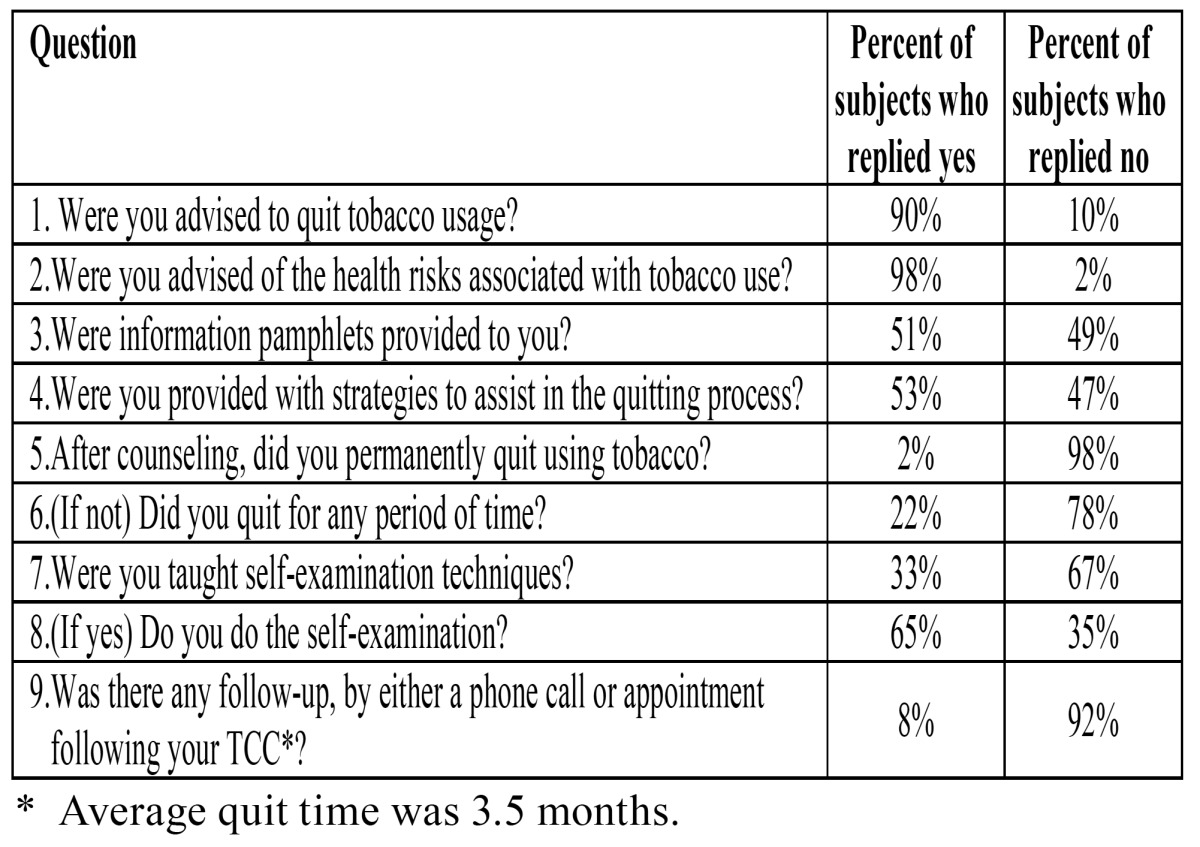
Tobacco cessation counseling by dental hygienists. – Responses to survey questions among 132 dental patients (39).

## Conclusions

Particularly risk factor reduction and perhaps also early detection of oral cancer and precancer will decrease the morbidity and mortality of oral cancer.

There should be ways to increase the awareness of the public about oral cancer and precancer, including knowledge about the risk factors. At present, the results of mouth self-examination are somewhat disappointing. Mass screening for oral cancer is regarded not to be cost-effective. Instead, screening of selected high risk groups may be worth to perform in an attempt to diagnose oral cancer in an earlier tumor stage, thereby allegedly reducing the morbidity and mortality rate.

Dentists and physicians should be continuously advised to attend programs that are directed at the early detection of oral cancer and precancer.
